# Exceptional molecular and coreceptor-requirement properties of molecular clones isolated from an Human Immunodeficiency Virus Type-1 subtype C infection

**DOI:** 10.1186/1742-4690-5-25

**Published:** 2008-03-07

**Authors:** Prasanta K Dash, Nagadenahalli B Siddappa, Asokan Mangaiarkarasi, Aruna V Mahendarkar, Padmanabhan Roshan, Krishnamurthy Kumar Anand, Anita Mahadevan, Parthasarathy Satishchandra, Susarla K Shankar, Vinayaka R Prasad, Udaykumar Ranga

**Affiliations:** 1 Molecular Virology Laboratory, Molecular Biology and Genetics Unit, Jawaharlal Nehru Centre for Advanced Scientific Research, Bangalore, India; 2 Department of Neurology, National Institute of Mental Health and Neurosciences, Bangalore, India; 3 Department of Neuropathology, National Institute of Mental Health and Neurosciences, Bangalore, India; 4 Department of Microbiology and Immunology, Albert Einstein College of Medicine, Bronx, NY, USA; 5 Dana-Farber Cancer Institute, Harvard Medical School, 44 Binney Street, JFB-809, Boston, MA 02115-6084, USA

## Abstract

**Background:**

The pathogenic significance of coreceptor switch in the viral infection of HIV-1 is not completely understood. This situation is more complex in subtype C infection where coreceptor switch is either absent or extremely rare. To gain insights into the mechanisms that underlie coreceptor requirement of subtype C, we screened several primary viral isolates and identified a clinical sample that demonstrated a potential to grow on standard T-cell lines with no detectable CCR5 expression. The subject was diagnosed with HIV-1 associated dementia in the absence of opportunistic infections of the brain. To isolate molecular clones from this virus, we devised a novel strategy based on anchor primers that target a sequence in the reverse transcriptase, highly conserved among diverse subtypes of HIV-1.

**Results:**

Using this strategy, we isolated 8 full-length molecular clones from the donor. Two of the eight molecular clones, 03In94_D17 and 03In94_D24, (D17 and D24) generated replication-competent viruses. Phylogenetic analysis of the full-length viral sequences revealed that both clones were non-recombinant subtype C viruses. They contain intact open reading frames in all the viral proteins. Both the viral clones are endowed with several unique molecular and biological properties. The viral promoter of the clones is characterized by the presence of four NF-kB binding elements, a feature rarely seen in the subtype C HIV-1 LTR. Interestingly, we identified the coexistence of two different forms of Rev, a truncated form common to subtype C and a full-length form less common for this subtype, in both proviral and plasma virus compartments. An exceptional property of the viruses, atypical of subtype C, is their ability to use a wide range of coreceptors including CCR5, CXCR4, and several others tested. Sequence analysis of Env of D17 and D24 clones identified differences within the variable loops providing important clues for the expanded coreceptor use. The V1, V2 and V4 loops in both of the molecular clones are longer due to the insertion of several amino acid residues that generated potential N-linked glycosylation sites.

**Conclusion:**

The exceptional biological and molecular properties of these clones make them invaluable tools to understand the unique pathogenic characteristics of subtype C.

## Background

Of the various subtypes of Human Immunodeficiency Type I (HIV-1) and their recombinant forms, the subtype C strains are responsible for the rapidly expanding epidemics in the most populous nations such as India, China, Sub-Saharan African countries and southern Brazil. [1]. More than half the new HIV-1 infections in the world [2] and nearly 99% of the infections of India [3] are due to subtype C. During the past decade, the infection incidence of subtype C in southern Brazil reportedly increased from 3% to 30% [4] and eventually to 45% to 48% [5,6]. In view of the rapid global expansion of subtype C, this viral subtype is believed to have evolved to be less pathogenic to the human host [7]. The expansion dynamics of viral subtypes might be governed by cis-regulatory elements including the promoter sequences, as well as viral proteins with regulatory, structural or accessory functions. Subtype C strains of HIV-1 are endowed with unique biological properties including the infrequent coreceptor switch [8,9]. New viral infections are initiated by viral strains that require the coreceptor CCR5 on the target cell (R5 strains), regardless of the subtype nature [10,11]. However, towards the later stages of the infection, nearly half of subtype B strains switch their coreceptor usage to CXCR4 (X4 strains) [12,13]. The emergence of X4 strains coincides with disease progression to AIDS [14] and as a consequence, X4 viruses are believed to be more pathogenic than R5 strains [15,16], a controversial proposition [17,18]. Importantly, coreceptor switch from CCR5 to CXCR4 is less common in subtype C infection [8,9,19-22], the pathological significance of which, is not understood. In subtype C, presence of a small minority of viruses that can use CXCR4 has, nevertheless been documented [8,21,23-26]. The actual incidence of CXCR4 using subtype C strains in natural infection, the time of their appearance in the disease progression and the relevance of their emergence have not been elucidated.

The current methods of viral molecular cloning permit isolation of only near full-length clones that are replication-defective. Molecular repair will be necessary to generate replication-competent viruses from such clones, a procedure time consuming and laborious. Given that only three subtype C infectious molecular clones are available presently [27-29], with an objective to isolate molecular clones of replication-competent viruses from subtype C infection, especially from individuals with HAD, we developed a cloning strategy that obviates a need for molecular repair. We report here, the isolation of several full-length molecular clones from the peripheral blood of an HIV seropositive subject with HAD from India. A biological virus generated from this donor demonstrated a potential to grow in standard T-cell lines suggesting expanded coreceptor use, prompting us to generate molecular clones from this virus.

## Results

### Isolation of molecular clones from an Indian donor with HAD

**Figure 1:**
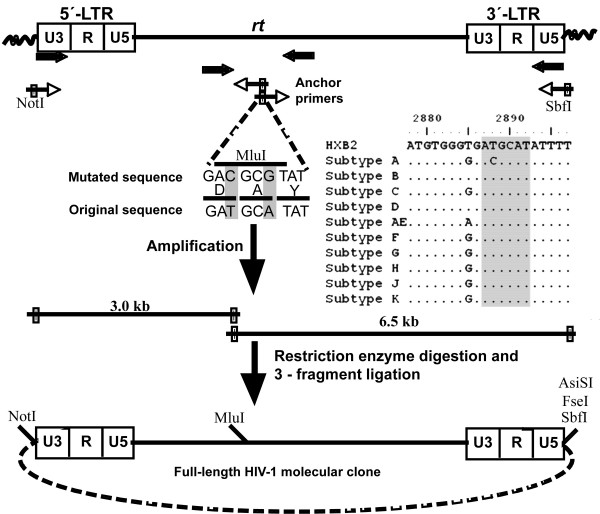
Schematic representation of the anchor primer-based cloning strategy for HIV-1: An integrated provirus is depicted at the top of the cartoon with wavy lines representing the cellular genomic DNA. The figure is not drawn to the scale. The U3, R and U5 regions of both the LTRs are delineated. Arrows represent primer location and the orientation. The open arrows are used for the first-round and the filled arrows for the second round amplifications. Note that only the second-round primers contain restriction sites for cloning (filled boxes). The anchor primers, in both the orientations, are complementary to a highly conserved sequence within *rt*. The 6 nucleotide sequence into which the MluI site is engineered (the inset) and the flanking sequences where the anchor primers anneal (not completely shown) are highly conserved among diverse viral subtypes of HIV-1. Dots indicate sequence homology. The amino acid sequence is shown in the single letter code. The coordinates are as in HXB2 molecular clone (accession number K03455). The sequence into which MluI site was engineered is highlighted by shading. The complete viral genome is amplified in two unequal fragments and cloned directionally into a vector in a single ligation reaction to generate full-length molecular clones. Additional restriction sites, all 8 bp recognition sequences that can be used for the viral cloning are also shown.

Virus isolated from the plasma of an Indian donor with HAD (BL94/03) proliferated on HOS-CCR5, HOS-CXCR4 cells and certain T-cell lines that lacked detectable surface expression of CCR5 suggesting expanded coreceptor use (see Additional file 1). With an objective to isolate infectious molecular clones from this subject, we developed a cloning strategy that could be applicable universally to all HIV-1, HIV-2 and SIV subtypes. The strategy presented here permits amplification of full-length molecular clones from integrated proviral DNA, obviating a need for molecular repair strategy to generate infectious viruses. The strategy depends on the use of a pair of anchor primers in the reverse transcriptase (RT) gene of HIV-1 (Figure 1). The anchor primers permit amplification of the provirus from genomic DNA in two independent but complementary fragments which, when assembled, can generate full-length viral clones. We engineered a restriction enzyme site for MluI in the anchor primers by changing two base pairs in two adjacent codons without changing the amino acid sequence of RT (Figure 1). The region of RT selected for anchor primer binding and MluI engineering is highly conserved among all the major viral subtypes of HIV-1 (Figure 1, inset) therefore, viral genetic heterogeneity is not likely to influence the cloning efficiency. Importantly, MluI site is significantly underrepresented in diverse subtypes of HIV-1 (Table 1). For instance, MluI was found only in 2 of the 162 near full-length subtype C sequences available at the Los Alamos HIV sequence database. MluI is also significantly underrepresented in other subtypes of HIV-1 and HIV-2 (Table 1). Two external primers were designed to bind the 5' and 3' ends of the 5' and 3' LTRs, respectively. The external primers contained NotI and SbfI sites, respectively, to permit directional cloning of the amplified fragments (Figure 1). Both of these enzymes recognize 8 bp sequences that are CG-rich, unlike the HIV-1 genome, which is AT-rich. Additionally, sequence analysis confirmed absence of NotI recognition motifs and under-representation of SbfI in diverse viral subtypes (Table 1). Using the two primer pair combinations (a) NotI forward primer and MluI reverse anchor primer and (b) MluI forward anchor primer and SbfI reverse primer, HIV-1 provirus can be amplified in two fragments, the 5' and 3' fragments of 3.0 and 6.5 kb length, respectively.

**Table 1:** Frequency of restriction enzyme sites in HIV

**Type**	**Group**	**Subtype**	**N**	**NotI**	**MluI**	**SbfI**	**FseI**	**AsiSI**
**HIV-1**	**M**	**A**	86	0	0	7	0	0
		**B**	211	17	4	99	0	0
		**C**	425	8	3	95	0	0
		**D**	61	0	1	46	0	0
		**A/E**	92	0	1	49	0	0
		**F**	20	0	2	12	0	0
		**G**	130	0	3	10	0	0
		**H**	100	0	1	3	0	0
		**J**	73	0	0	1	0	0
		**K**	23	0	0	13	0	0
	**N**		29	0	0	2	0	0
	**O**		143	0	3	30	0	0
**HIV-2**			18	1	1	2	0	0

N = Number of sequences analyzed.

We applied the above cloning strategy to genomic DNA extracted from the peripheral blood of the donor BL94/03. Genomic DNA extracted on day-7 of the viral coculture, where the p24 concentration in the medium was the highest, was used for the amplification of the provirus. Both of the amplified fragments were restriction digested and simultaneously cloned into a vector engineered to contain NotI and SbfI. We identified 8 full-length molecular clones from a single ligation reaction.

### Two of the 8 viral molecular clones are infectious

**Figure 2:**
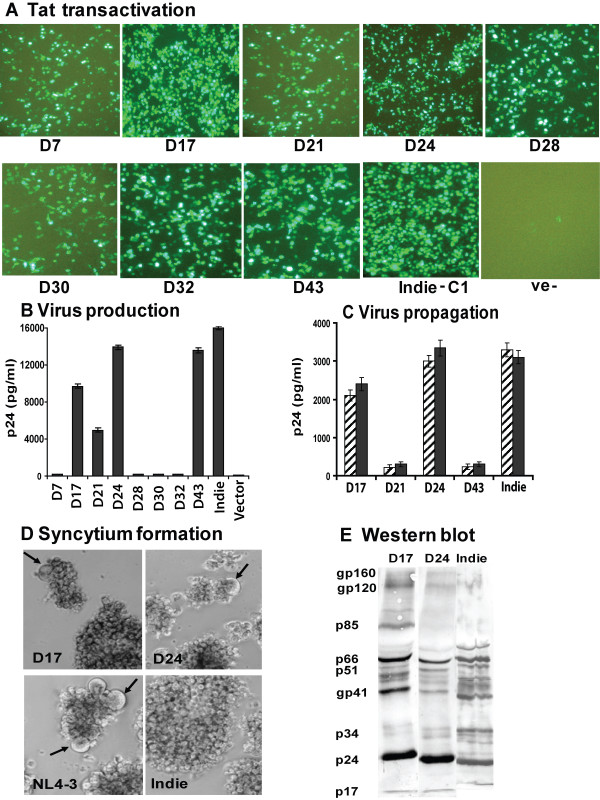
Analysis of viral functions from the viral molecular clones. **(A) **Ability of the molecular clones to encode functional Tat protein. The 8 molecular clones were individually cotransfected into HEK293 cells along with a reporter construct expressing EGFP under the control of HIV-1 subtype C LTR. Subtype C reference molecular clone, Indie-C1, serves as a positive control and the parental cloning vector as a negative control. GFP+ cells were documented 48 h following the transfection. **(B) **Virus production from the molecular clones. Spent media from the above cultures were monitored for p24 using a commercial antigen-capture ELISA. Note that only 4 out of 8 clones express p24 indicating virus production. **(C) **The ability of virus produced by the molecular clones to propagate through the PBMC in culture. Mitogen-activated PBMC from two different healthy donors (striped and filled bars) were infected with the virus produced from the 4 molecular clones that secreted p24 in 'B' above. Viral stocks equivalent of 10 ng of p24 were used for the infection. Following 4 h of incubation, the residual virus was removed by washing, the cells were cultured and production of p24 into the culture medium was monitored using an antigen-capture assay. Data for day-7 have been presented. Note that only two molecular clones, D17 and D24, were found to be infectious. **(D) **Syncytium formation by the infectious molecular clones in MT2 cells. Both D17 and D24 induced syncytium formation (arrows) in MT2 cells within a couple of days, same as NL4-3. **(E) **Analysis of the viral antigens by western blotting. HEK293T cells were transiently transfected with D17, D24 or Indie-C1 molecular clones. Cell-free culture supernatants were collected 72 h post-transfection and subjected to high-speed centrifugation to pellet the virus. Viral pellets were directly suspended in lysis buffer and the viral antigens were separated on a 6 to 15% gradient SDS-PAGE gel. Proteins were transferred to PVDF membrane and analyzed by immunoblotting using pooled sera derived from individuals infected with HIV-1 subtype C. Predicted viral antigens are indicated.

Next, we tested for the expression of Tat protein during virus replication. We cotransfected HEK293T cells with each of the 8 HIV molecular clones separately, with a reporter construct expressing GFP under the control of HIV-1 LTR. All 8 molecular clones as well as the reference subtype C clone Indie-C1 [27] upregulated the expression of GFP suggesting the production of functional Tat (Figure 2A). Production of Tat protein in an infected cell signals that a successful infection has been established including entry, reverse transcription, nuclear transport, integration, transcription and splicing. To detect active virus replication, we determined the quantity of p24 in the supernatant, as a measure of particle release, at multiple time points. On day-3, only 4 of the 8 molecular clones (D17, D21, D24 and D43) produced p24, while others (D7, D28, D30 and D32) failed to make viral protein, suggesting defects in late events (Figure 2B). To evaluate the replication competence of the four clones that produced p24, we added 10 ng equivalent of p24 from the culture supernatant to mitogen-activated donor PBMC and monitored p24 synthesis up to day-22. Two of the four molecular clones (D17 and D24) readily replicated in the PBMC and secreted p24 into the medium starting from day-2, the concentration of which, peaked on day-7. The other two clones (D21 and D43) failed to replicate on PBMC (Figure 2C). Interestingly, unlike typical subtype C viruses, both D17 and D24 generated morphologically distinct syncytia in MT-2 cells (Figure 2D) and PBMC (not shown) that were numerous, remained small and regular in shape. A Western blot analysis of the virion proteins using seropositive donor sera revealed that all major viral proteins could be detected in virions produced by both viral clones. (Figure 2E). The defective nature of a majority of the viral clones, 6 out of 8, is rather expected, given that these are products of reverse transcription, an error prone process. In a large number of previous studies, where several viral clones were isolated, a large majority of the clones were found to be defective [30-34].

### Sequence analysis of the full-length genomes

**Figure 3:**
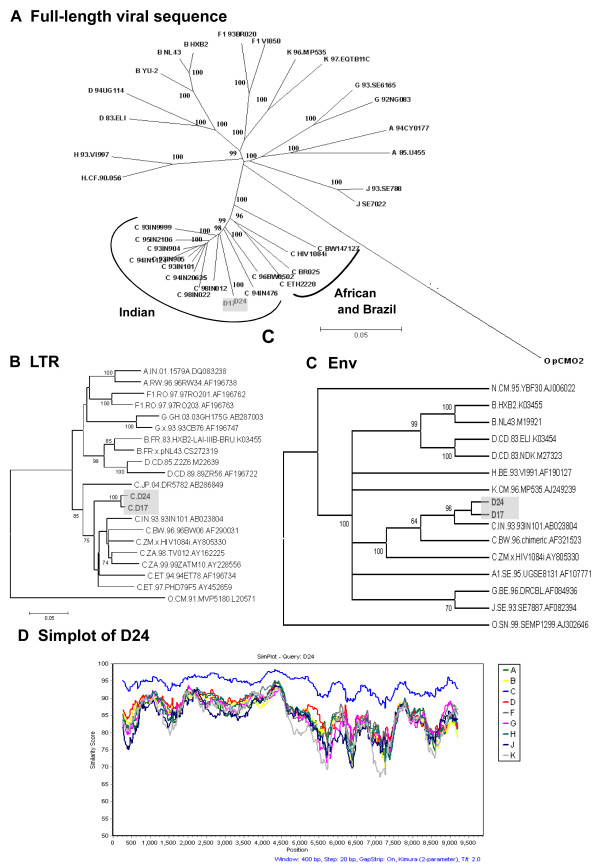
Sequence analysis of the molecular clones D17 and D24. **(A) **Phylogenetic relationship of the molecular clones. Full-length sequences of D17 and D24 (shaded) were compared with representative HIV-1 sequences of subtypes A, B, C, D, F, G, H, J and K. subtype C sequences from India were also included in the analysis. A neighbor-joining tree was constructed on the basis of the hidden Markov model nucleotide alignment of full-length HIV-1 genomes. Subtype O pCMO2 sequence was used as the outgroup. Horizontal branch lengths are drawn to scale with the scale bar representing 0.05 nucleotide substitution per site whereas the vertical separation is only for clarification. Figures along the branches indicate the bootstrap values that support branching, out of a total of 1,000 resamplings. The reference sequences for different HIV-1 groups and subtypes were obtained from the HIV sequence database[97]. Phylogenetic trees of D17 and D24 based on the LTR **(B) **and the Env **(C) **sequences. Phylogenetic trees were constructed from full-length LTR and Env nucleotide sequences by using the neighbor-joining method. Major subtypes of HIV-1 group M were used as reference sequences. Trees were rooted by using subtype O strains as outgroups. Accession numbers for each of the references sequences used in the above plots could be obtained by using the name of the viral strain shown **(D) **Plot of similarity of D24 to a set of reference subtype genomes. Analysis was performed with the SimPlot program using a window size of 400 nucleotides and a step size of 20 nucleotides. Positions containing gaps were excluded from the comparison. The *x*-axis indicates the nucleotide positions along the sequence alignment. The *y*-axis denotes the distance between compared sequences plotted at the midpoint of the 400-nucleotide window. Identical results were obtained with the clone D17 (data not presented).

The complete nucleotide sequence of both D17 and D24 clones was determined. The proviral DNAs of D17 and D24 contained 9,831 and 9,830 bp, respectively, from the 5' LTR to the 3' LTR. The reading frames of all the structural, regulatory and accessory proteins are preserved in both D17 and D24. We determined the phylogenetic relationship of D17 and D24 sequences in their entirety (Figure 3A), as well as in the *LTR *(Figure 3B), *env *(Figure 3C) and other individual viral genes (data not shown). In these analyses, both of the viral molecular clones tightly clustered with subtype C reference sequences. Within the subtype C cluster, they are closely associated with the Indian reference strains, but not with those of Africa or Brazil, confirming their Indian origin (Figure 3A). To ascertain their non-recombinant nature, we performed genome-wide sequence comparison of both of the clones to non-recombinant subtype-representative reference clones (Figure 3D). In summary, these analyses identified both D17 and D24 to be non-recombinant subtype C viruses.

### Viral enhancer contains four NF-κB sites

The molecular clones, D17 and D24, are both endowed with several molecular features, some of which are representative of subtype C, while others are unique. The unique properties include the presence of four putative NF-kB sites in the LTR, expression of non-truncated form of Rev and extended V1/V2 domains in Env.

**Figure 4:**
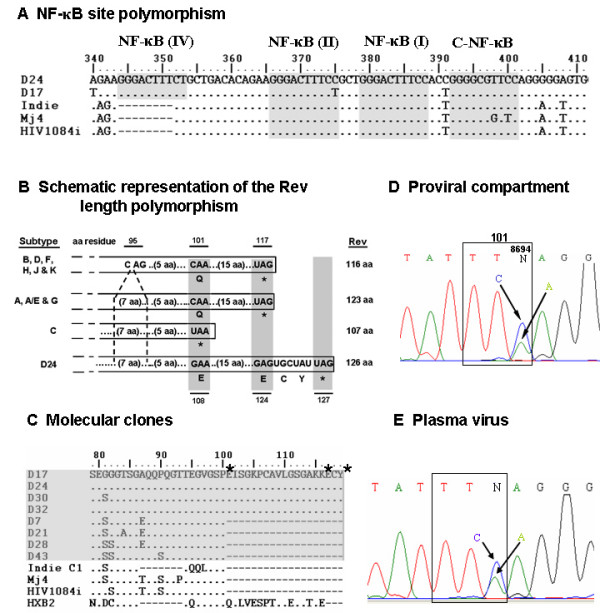
Unique molecular features identified in D17 and D24. **(A) **κB-site polymorphism in the viral enhancer. The nucleotide sequences within the enhancer region of D17 and D24 have been aligned with the three available infectious subtype C molecular clones using Clustal W program. Dashes represent gaps in the sequence and dots sequence homology. The sequence coordinates are as per the D24 molecular clone (accession number EF469243). The three canonical NF-κB sites and the upstream additional κB-element are highlighted. Note that at position 398, the molecular clone MJ4 (AF321523) contains a T to G variation in the C-NF-κB site that is expected to make this element non-functional. **(B) **Schematic representation of Rev expression variation in diverse subtypes of HIV-1. The C-terminus of D24 Rev is compared with that of diverse viral subtypes. The amino acid residues are represented in the single letter code. The coordinates of the amino acid residues are as per the HXB2 molecular clone (at the top). Subtypes A, A/E, G and C strains contain a 7 amino acid insertion at the residue 95 of HXB2 equivalent as shown. At residue 101, only subtype C strains typically contain a stop codon (highlighted by shading) that is non-functional in D24. A second stop codon (highlighted) at position 117 that terminates Rev in all non-subtype C strains is also non-functional in D24. Rev translation in D24 is terminated by a third stop codon (highlighted) located after a tyrosine residue at position 126. The expected size of the Rev proteins from different subtypes is shown on the right hand side. Stop codons are indicated by an asterisk; aa, amino acid. **(C) **Presence of two different variant forms of Rev in the molecular clones. Amino acid sequences at the C-terminus of all the 8 HIV-1 molecular clones reported in the present work (shaded) and the three subtype C infectious clones are aligned. Rev sequence of HXB2 is also included for comparison. Dots represent sequence homology and dashes gaps. The sequence coordinates are as per HXB2. Asterisks indicate putative stop codons (see 'B' above). Coexistence of two different variant forms of Rev in the donor provirus **(D) **and plasma virus **(E)**. Using a pair of primers, Rev exon-2 was amplified in a DNA PCR using genomic DNA extracted from the PBMC of the donor BL94. Alternatively, viral RNA was extracted from the plasma sample, reverse transcribed and the exon-2 of Rev was amplified using template-specific primers. The sequence information of the anti-sense strand was obtained directly from the PCR or RT-PCR amplicons. The codon of the amino acid residue 101 is boxed. Presence of both 'C' and 'A' at position 8695 (amino acid residue 101 of Rev) on the anti-sense strand confirmed the simultaneous existence of two different forms of Rev in the provirus as well as in the plasma virus.

An analysis of the LTRs using the motif search software [35] confirmed the presence of several potential transcription factor binding sites (TFBS) in the LTR of both the clones. These sites include the AP1, NF-Y, NF-AT, USF, LEF-1, p54 sites and several others in the modulatory region, NF-κB sites in the enhancer region and Sp1 sites and the TATA box in the core promoter. The enhancer element of D24 is characterized by a total of 4 NF-κB binding motifs (Figure 4A). Two of the threee κB-motifs (I and II) are identical to each other in sequence (GGGACTTTCC) that are the same as found in the subtype B LTR. The third κB-motif, proximal to the Sp1 sites, however, is unique in the sequence content and differs from the other two at two sites (GGGGCGTTTCC, differences underlined). As the latter is found only in subtype C LTR (C-LTR) we refer to this motif as C-NF-κB site. Importantly, in D24, in addition to the three canonical κB sites, we identified an additional (fourth) κB-motif 12 bp upstream of the κB region. The sequence motif of this κB site (GGGACTTTCT) deviated from the canonical HIV-1 κB sequence at position 10 where a 'T' is substituted for a 'C' (Figure 4A). Further, although the enhancer of D17 is characterized by the presence of four κB-sites like that of D24, additional level of κB polymorphism was evident. In this clone, the κB II site was found to be identical to the κB IV site. Consequently, D17 contains an authentic C-κB site, a canonical κB-site and two variant κB elements. Given that several cellular κB-sites deviate from the consensus motif, yet bind specific Rel-family members [36], the small deviation we observed at position 10 of the κB-like motifs in D17 and D24 may not have significant impact on the LTR function.

### D17 and D24 encode the longest Rev consisting of 126 amino acid residues

Rev protein of HIV-1 plays a key role in viral replication by regulating the nuclear export of unspliced viral mRNAs [37]. Both D17 and D24 contained a Rev open reading frame encoding 126 amino acid residues as a result of amino acid-substitution for two of the natural stop codons (Figure 4B). In these clones, the first stop codon (UAA) at position 101 (HXB2 coordinate) that is functional in all of the subtype C viruses has been substituted by 'GAA' coding for a glutamic acid residue. The second stop codon (UAG) in frame at position 117 (HXB2 coordinate) and functional in all non-C subtypes has also been replaced by 'GAG' coding for a glutamic acid residue. In both D17 and D24, translation of Rev is terminated at a third stop codon (UAG) that is located 6 bp downstream from the second stop codon, inserting two additional amino acid residues, cysteine and tyrosine, into the protein at the C-terminus (Figure 4B). As a consequence of these variations, Rev in D17 and D24 consists of a total of 126 amino acid residues, but not 107 residues typical for subtype C strains.

### Coexistence of two length-isoforms of Rev in the donor viral pool

To examine if the non-truncated Rev is the characteristic feature of the donor from whom the virus was isolated, we determined the Rev sequence of the 6 other non-infectious molecular clones that we isolated in this work (Figure 1). Surprisingly, we identified that only 4 of the 8 molecular clones (D17, D24, D30 and D32) contained a full-length Rev, while the other 4 (D7, D21, D28 and D43) possessed a truncated Rev (Figure 4C). The stop codon at position 101 was functional in the latter set of the viral clones yielding truncated Rev. To ensure that the presence of the non-truncated Rev in half of the viral clones was not a cloning artifact, we amplified the Rev gene from the donor genomic proviral DNA and directly sequenced the amplified product. At position 8695 of D24, we identified the presence of both 'G' and 'T' nucleotides (Figure 4D) confirming the coexistence of the length-isoforms of Rev in the integrated provirus. Since proviral DNA represents only the archived, but not the actively proliferating virus in the blood, we determined the sequences of Rev in the plasma virus. Total RNA was isolated from the original plasma sample and using sequence specific primers exon-2 of Rev was reverse transcribed, amplified and the sequence information was obtained directly from the PCR amplicon. In the viral cDNA, presence of both 'G' and 'T' nucleotides at position 8695 was evident (Figure 4E), confirming the presence of two different viruses also in the plasma, one expressing the truncated Rev and the other the full-length Rev.

### Other features of D17 and D24 common to subtype C viruses

Subtype-specific variations have been identified within the pyrimidine-rich 3 bp bulge of TAR. Subtypes A and D contain a 'UUU' at position 22 to 24 of the TAR from the transcription start site that constitutes the bulge. In subtype E, the bulge consists of only two bp of 'UC'. In other subtypes, a 'UCU' is found at this position. Surprisingly, in D24 we found 'UCC' constituting the bulge of the TAR (data not presented) a variation that has not been reported previously to the best of our knowledge. The Tat protein in D17 and D24 is encoded by two exons and consists of 101 amino acid residues. Of the 7 signature amino acid residues that we previously identified in subtype C Tat [38], three are preserved in D17 and D24 (S31, S57 and E63). Two important subtype-specific properties, the substitution of cysteine at position 31 by serine and the lack of a RGD motif in exon-2 are preserved in both clones. The Vpu protein of subtype C viruses is characterized by two motifs at either end of the protein. A highly variable 5 amino acid sequence motif is seen at the N-terminus of subtype C Vpu that is expected to extend the membrane-spanning domain [30]. We found an insertion of YKLTV in both D17 and D24 at this location. A four amino acid motif of LRLL, which is found at the C-terminus of subtype C Vpu [39], is also preserved in both of the clones. Both D17 and D24 encode an Env of 875 amino acid residues that are almost identical except for variations at 3 positions. The Env contains a signal peptide, gp120 and gp41 of 27, 494 and 354 amino acid residues, respectively. The motif, REKR, the cellular furin protease cleavage site at the junction of gp120 and gp41 is preserved. gp120 and gp41 of both the clones contain 31 and 4 putative N-glycosylation sites, respectively, including the 'sequon' at 451 which is unique for subtype C isolates of HIV-1 [40]. Additionally, both clones lack the N-glycosylation site (position 308 in D17 and D24) just upstream of V3 loop, as is typical of subtype C isolates [40]. Both the clones contain all three signature amino acid variations, (E340N, A350R and E429K) of Indian subtype C gp120 [41].

### Replication kinetics of viruses produced by molecular clones in different cell types

**Figure 5:**
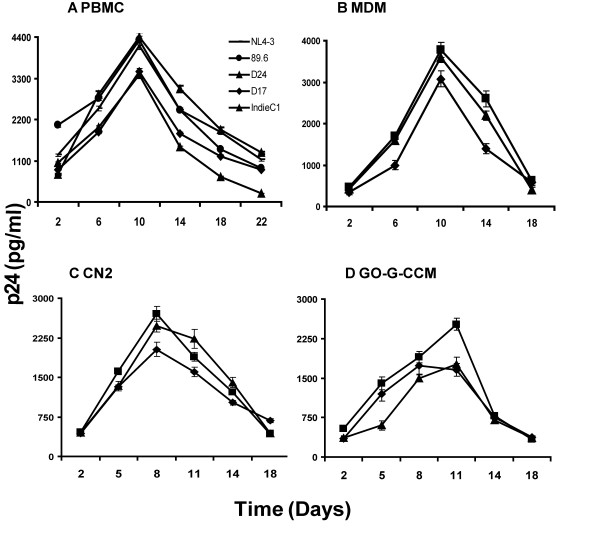
Replication kinetics of subtype C molecular clones in different cell types. CD8-depleted and mitogen-activated PBMC **(A) **monocyte-derived macrophages **(B) **CN-2 cells, an *Herpesvirus saimiri *immortalized cell line **(C) **and a human brain astrocytoma cell line, GO-G-CCM **(D) **were infected with 500 TCID_50 _units of different viral molecular clones. While proliferation of D17and D24 was compared with that of Indie-C1 in all the cell types, in PBMC alone, subtype B standard clones NL4-3 and 86.9 (open symbols) were also used. Viral replication was monitored by quantifying p24 levels in the culture medium at defined time points using a commercial antigen-capture ELISA. The data presented are representative of three independent experiments. The data are presented as mean quantity of p24 released in triplicate wells ± 1 SD.

We examined the growth kinetics of D17 and D24 in different mammalian cells and cell lines including primary blood mononuclear cells (PBMC), monocyte-derived macrophages (MDM), an astrocytoma cell line GO-G-CCM and *Herpesvirus saimiri *transformed T-cells, the CN-2 cells (Figure 5). Monocytes were isolated from fresh blood using two rounds of density gradient centrifugation [42]. CN-2 cells represent heterologous, CD8-devoid, immortalized PBMC population of a seronegative donor that can support virus proliferation efficiently, as reported previously [43]. Both D17 and D24 and three different reference viral clones readily proliferated in PBMC, monocytes, CN-2 and the astrocytoma cells. The presence of p24 was detectable in the media starting from day-2 of culture that reached peak levels around day 10 or 11 in the PBMC, monocytes and the astrocytoma cells. In CN-2 cells, the peak was observed a little earlier, on day 8. By and large, the growth kinetics of both D24 and Indie-C1 appeared comparable in all cell lines tested in terms of p24 levels and the time needed to reach the peak levels. The growth kinetics of D17, although, followed the same profile; the magnitude of p24 produced was significantly lower in comparison to that of D24 in all the four types of cells. Surprisingly, the replication kinetics of both D17 and D24 and Indie-C1 are comparable in activated PBMC and the astrocytoma cell line GO-G-CCM. Astrocytoma cells normally do not support viral proliferation to the same extent as PBMC. Individual cell lines could differ significantly in several biological properties, hence it is necessary to examine viral proliferation in diverse cell lines of neuronal origin and importantly in primary fetal astrocytes.

### Expanded coreceptor use of D17 and D24

**Figure 6:**
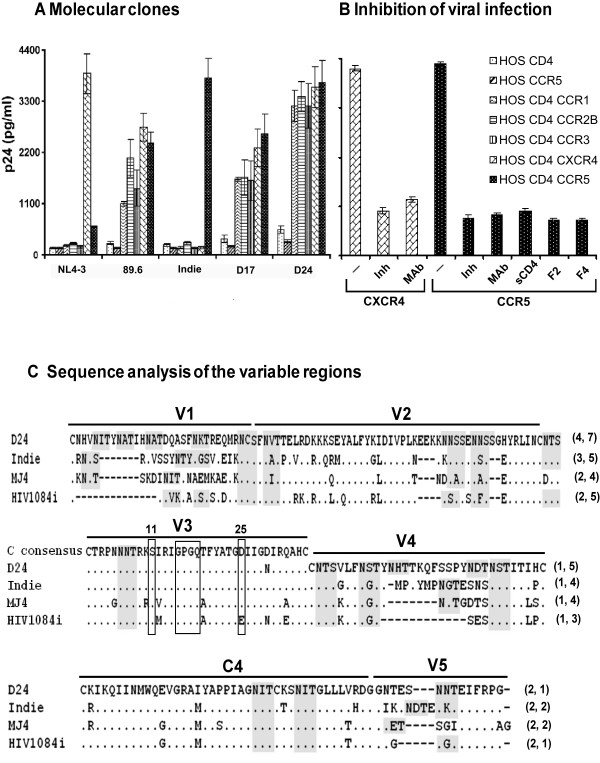
Coreceptor phenotyping of the viral molecular clones. **(A) **Profile of coreceptor use in HOS cell lines. HOS cell lines expressing CD4 and one of the several coreceptors including CCR1, CCR2B, CCR3, CXCR4 and CCR5 or CD4 alone or CCR5 alone were tested for viral infectivity. These cell lines were infected overnight with one of the viral molecular clones (89.6, NL4-3, Indie-C1, D17 and D24), residual virus was washed off and the cells were incubated up to 14 days. Secretion of p24 into the medium was monitored every third day using a commercial kit and data for day-7 are presented here. Data are representative of at least four independent experiments and presented as mean quantity of p24 released in triplicate wells ± 1 SD. Identical results were obtained at other time points. **(B) **Inhibition of the viral infection using receptor-specific inhibitors. Target cells, HOSCD4CXCR4 and HOSCD4CCR5, were infected with the viruses in the absence or presence of one of the inhibitors (Inh) at appropriate concentration. CXCR4 inhibitor bicyclam JM-2987 at 60 nM, CCR5 inhibitor TAK-779 at 20 μM, soluble CD4 (sCD4) at 1 μg/ml and two different anti-human CD4 monoclonal antibodies F2 and F4 at 0.5 μg/ml were used. In a similar fashion, we used coreceptor-specific monoclonal antibody pools (Mab), 0.5 μg of each antibody, to block viral infection, antibodies # 1012 and # 1009 for CXCR4 (Pro-science, Poway, CA) and antibodies #182 (R&D systems) and #624085 (BD Biosciences) for CCR5. The residual virus and the inhibitors were removed after overnight incubation and the p24 production was monitored in the culture supernatants. Data for only D24 virus on day-7 post-infection are presented here and identical results were obtained at other time points and with other viruses. **(C) **Amino acid sequence comparison of the gp120 extra-cellular variable loops and the C4 domain. The amino acid sequences of D24 env loops are aligned with those of the three available subtype C infectious molecular clones. Dots indicate sequence homology and dashes gaps. For the V3 loop, subtype C consensus sequence is presented at the top and the positions 11 and 25 and the 'GPGQ' motif have been highlighted using open boxes. Putative N-linked glycosylation sites have been highlighted using shaded boxes. Figures in the parentheses indicate the number of potential glycans in respective loops. Viral clone D17 is not shown here as the amino acid sequences of D17 and D24 in gp120 differ from each other at only one position.

Since the biological virus of the donor originally proliferated on standard T-cell lines, we examined the coreceptor requirement of the molecular clones isolated. We used HOS cell lines expressing CD4 and one of the several coreceptors including CCR5, CXCR4, CCR1, CCR2B or CCR3 in these assays. In addition to D17 and D24, we used reference HIV-1 molecular clones with known coreceptor use profile as controls, Indie-C1 for CCR5, NL4-3 for CXCR4 and 89.6 for both the coreceptors. Cells were infected with 500 TCID_50 _units of the viral stocks and the amount of p24 secreted into the medium was determined at different periods (Figure 6A). The reference molecular clones Indie-C1 and NL4-3 used only CCR5 and CXCR4, respectively, for target cell infection and failed to use other coreceptors. Virus 89.6, in contrast, used all the coreceptors tested. Significantly, both D17 and D24 used all five coreceptors nearly with equal efficiency, although the clones appeared to prefer CCR5 and CXCR4 over others. The clone D17 released significantly low quantities of virus as compared to D24 with any of the 5 coreceptors tested suggesting that the differential replication potential of this clone is possibly not because of a bottleneck at the cell entry level (data not shown). To confirm receptor- and coreceptor-dependent nature of cell infection of the viruses, we used small molecule or monoclonal antibody inhibitors, at appropriate concentrations, to block viral infection (Figure 6B). We observed 85–90% inhibition in D24 viral infection in the presence of two different anti-human CD4 monoclonal antibodies (generated in-house), soluble CD4, small molecule inhibitors against the coreceptors (TAK-779 and JM-2987 for CCR5 and CXCR4, respectively) or monoclonal antibodies to the coreceptors. We obtained similar levels of reduction in virus titers also with D17 and other reference viruses in the presence of appropriate inhibitors (data not shown).

### V3 loop of both D17 and D24 represents a typical R5 consensus amino acid sequence

To understand the basis for the expanded coreceptor use of the molecular clones D17 and D24, we first examined the molecular features of the V3 loop in the env as the V3 loop significantly influences and determines coreceptor preference of HIV isolates [44]. The V3 loop of D17 and D24 contains 35 amino acid residues that are representative of CCR5-using subtype C consensus sequence (Figure 6C). All the molecular features typical and suggestive of CCR5 use are significantly preserved in the V3 loop of D17 and D24. The presence of a neutral serine at position 11 and of an aspartic acid or aspargine with a negatively charged side chain at position 25 is suggestive of CCR5 use [45,46]. Both the clones contain a serine and a glutamine at these positions, respectively. The crown of V3 loop in both the clones contains GPGQ motif highly suggestive of CCR5 use [23]. The V3 loop of both the clones contained a net charge of +5. A net charge of +5 or less is indicative of CCR5 use [47]. The V3 loop lacks additions or deletions of amino acid residues. Furthermore, the N-glycosylation site within the loop is preserved, both properties being suggestive of CCR5 use. Molecular features of the V3 loop of D17 and D24 were suggestive of only CCR5 use. The clones, nevertheless, used a range of coreceptors strongly indicating that Env domains outside the V3 loop could be playing a critical role in determining coreceptor use. In subtype B infection, sequence variations within V1/V2, V4 and/or C4 have been reported to influence and modulate coreceptor preferences of the viral env although V3 primarily regulated the cell infection [48-52].

### Non-V3 loops of the env probably govern the expanded coreceptor use in D24

To determine if non-V3 env loops (V1/V2, V4 and V5) and/or C4 domain of D17 and D24 might possess unique molecular features suggestive of expanded coreceptor use, we aligned amino acid sequences of D24 with all the three available infectious reference molecular clones of subtype C that are known to use only CCR5 (Figure 6C). This analysis identified two molecular features unique to D24. First, D24 contained the largest V1, V2 and V4 loops when compared to the reference molecular clones as a consequence of amino acid insertions (Figure 6C). The V1 loop of Indie-C1 and MJ4 contained 23 residues while that of HIV108i contained the shortest loop with only 16 residues. In contrast, D24 contained a loop consisting of 30 amino acid residues. In a similar fashion, the V2 and V4 loops of D24 contained several stretches of amino acid insertion when compared to the reference molecular clones and the loops are larger by several amino acids. Second, inserted amino acid residues in D24, created additional putative N-linked glycosylation sites in these loops. For instance, insertion of a 7 amino acid stretch (ITYNATI) at the N-terminus of the V1 loop generated two potential gylcans (NITYNATI) in D24 (Figure 6C). An analysis for potential N-linked glycosylation sites in gp120 identified that D24 contained the largest number of glycans when compared to the other three subtype C reference molecular clones. While D24 contained a total of 31 such sites in gp120, the Indie-C1, MJ4 and HIV1084i clones contained 27, 23 and 24, respectively. The additional glycans in D24 are mapped to V1, V2 and V4 loops. Loss, rather than gain, of potential glycans in Env is, by and large, associated with expanded coreceptor use [53].

### Subtype C viruses from other donors with HAD use only CCR5

Clones D17 and D24 are exceptional, in that they are the first subtype C molecular clones to demonstrate expanded coreceptor use including that of CXCR4 and CCR5. Given that these clones originated from a donor with HAD, we sought to know whether expanded coreceptor use could be a general characteristic attributable to viruses derived from subjects with HAD. To answer this question, we isolated biological viruses from peripheral blood lymphocytes of two seropositive donors without neurological consequences (BL43/03, S53/03) and three subjects diagnosed with HAD (BL95/03, BL96/03, A43/05). Viral stocks were prepared and the TCID_50 _titer of each stock was determined as described in methods. All biological viruses, regardless of their HAD status, used only CCR5, but no other coreceptor, for cell infection (see Additional file 2A). D24 included in the assay used all the five coreceptors. Sequence comparison of the variable loops of the Env between D24 and the biological viruses broadly yielded similar results (see Additional file 2B). D24 contained the largest loops of V1, V2 and V4 as a result of multiple amino acid insertions, when compared to the biological viruses and also contained the largest number of N-linked glycosylation sites in gp120.

## Discussion

In this report, we described the construction, biological characterization and sequence analysis of infectious molecular clones of HIV-1 subtype C from one Indian subject with HAD. Although the resulting clones may not have originated from individual viruses, the original genetic diversity of the viral quasispecies is expected to be preserved, given that combination of two independent fragments is a stochastic process. To permit directional cloning of the amplified viral fragments, we selected restriction enzymes that are less represented in diverse viral subtypes (Table 1). The restriction enzyme site, MluI, engineered into the anchor primers did not change the original amino acid sequence in RT that is highly conserved among diverse viral subtypes (Figure 1). Unlike the anchor primers, the external primers binding the LTR are optimized to amplify subtype C sequences. The extreme ends of the viral LTR are adequately conserved within a subtype but not across diverse viral subtypes. It is, therefore, essential to optimize the external primers to amplify a given viral isolate based on the subtype nature.

All the previous studies, thus far, used virus isolates or clones of HIV-1 isolated from selected subjects to characterize the coreceptor requirement of the subtype C viruses [8,21,23-26,54]. Given the magnitude of genetic diversity present in the viral quasispecies in such preparations, molecular determinants responsible for governing coreceptor use is difficult to analyze. In view of this limitation of biological viruses, we developed the cloning strategy described here. Furthermore, this cloning scheme could be widely applied to other retroviruses including N and O groups of HIV-1, HIV-2 and SIV as MluI site is underrepresented in all these viruses.

In an attempt to delineate the viral determinants responsible for the low prevalence of HIV-associated dementia (HAD) in India caused by HIV-1 subtype C, we previously identified a natural variation at position 31 of subtype C Tat and demonstrated loss of monocyte chemotactic activity for subtype C Tat without loss in transactivation property [38]. We hypothesized that the Tat variation could be one of the factors for the low incidence of HIV-1 encephalitis and HAD in India. Apart from Tat, other viral proteins like Nef also have been shown to demonstrate chemokine-like properties [55,56]. The relative role of these as well as other viral proteins in modulating the leukocyte migration has not been systematically evaluated. This analysis is more relevant to subtype C strains considering the defective monocyte-chemokine nature of the C-Tat protein. To understand if functional redundancy exists between viral proteins in the chemokine-like property or the proposed loss in the chemokine property of subtype C Tat is compensated for by a variation co-selected in a different viral protein, it is important to subclone the viral proteins from a single replication-competent molecular clone rather than from genomic DNA, which is representative of quasispecies.

### κB site polymorphism in subtype C LTR

Of the several variations reported in the LTR of the viral subtypes, polymorphism within the κB binding sites of the C-LTR is quite unique. While a large number of C-LTRs contain three NF-κB sites in contrast to the usual two sites seen in several other subtypes [57-59], a significant minority contains a fourth motif that is a canonical κB site or κB-like element [58,60-62]. Previously we demonstrated that nearly 6% (35/608) of primary viral isolates of India contained length polymorphism in the enhancer [3,63]. The biological significance of the κB polymorphism in a significant number of C-LTRs has not been adequately evaluated. A gain of function has been ascribed to the C-LTR as it contains three κB elements as opposed to two in subtype B LTR. Extension of this logic further should make the D24 LTR a stronger viral promoter as it contains four κB sites. We tested this hypothesis in a variety of mammalian cells by placing a reporter gene under the control of several C-LTRs including that of D24. D24 LTR demonstrated the strongest reporter activity among all the LTRs tested [64] suggesting that the existence of a putative fourth κB-like site probably confers a strong transcriptional activity on this promoter. We will be extending these studies further to elucidate the importance of the κB polymorphism in subtype C promoter using D24 LTR.

### Truncated Rev appears to have a functionally important role in subtype C infection

Rev consists of two exons with exon-1 coding for 25 amino acid residues in all viral subtypes. In contrast, exon-2 exhibits extensive and often subtype-specific sequence variation as well as length polymorphism. A large majority of the strains of subtypes A, AE and G contain an exon-2 of 98 residues consequently making a mature Rev of 123 amino acids. Almost all the isolates of subtypes B, D and F and a large majority of H, J and K contain an exon-2 that is shorter by 7 residues, thereby making a mature Rev of 116 amino acids. Remarkably, a vast majority of subtype C isolates make the smallest Rev protein of only 107 residues as exon-2 in this subtype contains a premature stop codon leading to the deletion of the 16 amino acid residues at the C-terminus [30]. The termination is a result of a natural variation at the residue 101 of HXB2 equivalent where a 'CAA' coding for a glutamine is replaced by the termination codon 'UAA' (Figure 4B). Rev in Indie-C1, an infectious subtype C molecular clone, contains additional deletions and is shorter by as many as 25 amino acids compared to subtype B protein yet retains biological function [27]. Further, like subtype A, most of subtype C strains contain a stretch of 7 amino acid residues inserted into the codon 95 equivalent of HXB2 (Figure 4B).

The most unexpected finding of this study is the coexistence of two different forms of Rev in the proviral DNA and in the active plasma virus. We detected viruses containing both the truncated and the full-length forms of Rev at comparable levels in the patient DNA and plasma samples (Figure 4D and 4E). In a sequencing reaction, a minority variant population must be present at a frequency of at least 10–20% to generate a distinct signal [65]. Conspicuous signals at the position 8695 in the sequencing reaction not only confirmed the presence of both the Rev variants in the sample but also their existence in significant proportion in the viral population. Presence of Rev in two different forms in the same subject was unexpected and the relevance to disease progression to dementia is not understood. However, co-dominant expression of both the Rev variants (the absence of domination of one of these forms over the other) was surprising, given that such dominance is common for several viral variations. Importantly, coexistence of the Rev isoforms in the archived proviral compartment, at an equivalent proportion, points to the non-transient, prolonged and stable coexpression of these two Rev forms throughout the infection process of the donor. Prolonged and stable coexpression of these Rev forms is also strongly indicative of the functional importance of both the Rev forms that serve probably non-complementary and non-overlapping functions. Continued expression of truncated Rev, in the presence of full-length Rev, suggests independent functional importance of this Rev form for subtype C infection.

### Rev length variations in subtype C

Searching all the Rev sequences of subtype C in the Los Alamos database, representing global viral distribution, we identified length polymorphism in only 3% (4 out of 128) of the subtype C sequences analyzed. Interestingly, Rev in these subtype C variants, D17 and D24 included, codes for a protein containing 126 amino acid residues, not 123 as in other subtypes or 107 in subtype C, as a result of replacement of two stop codons. A glutamic acid residue replaces both the stop codons at positions 101 and 117 (HXB2 coordinate), by UAA to GAA and UAG to GAG substitutions, respectively (Figure 4B). Both the stop codons are replaced by single nucleotide variations that are highly conserved in subtype C suggesting a strong positive selection at these positions. Expression of gp41 in a different reading frame by the same nucleotide sequence adds an additional level of complexity for the analysis to identify the driving forces operating in the positive selection of these variations. A close examination of the sequences, however, indicates that selection of the first and second stop codon variations in subtype C is probably driven by Rev and gp41, respectively. The U8695G variation in these subtype C variant isolates, D24 included, at the first Rev stop codon, is a silent mutation that doesn't change the amino acid residue in gp41 (CUU and CUG both code for a leucine). Rev, therefore, could be the driving force for the positive selection of this variation. The T8742G variation, in contrast, substitutes a lysine residue for asparagine in gp41. The U8742G variation couldn't have been selected by Rev as in a large majority of subtype C isolates, the protein is terminated upstream at the position 8695 that constitutes the first stop codon. It is, therefore, reasonable to assume that the U8742G variation is positively selected by gp41 in subtype C viruses. In all the subtype C isolates with non-truncated Rev, D17 and D24 included, the protein consists of 126 amino acids as three additional residues (glutamic acid, cysteine and tyrosine) are added at the C-terminus as compared to subtype A that makes Rev of 123 amino acids. In a recent study on the Sydney blood bank cohort of long-term survivors, Churchill et al demonstrated the presence of longer forms of Rev in several of the subjects [66]. Rev in three of these subjects was longer by 3 residues whereas in one donor D36 by 13 residues. Importantly, the attenuated biological function of Rev in this subject was mapped to the 13 amino acid extension of Rev at the C-terminus. Based on this result, we are presently evaluating the influence of the C-terminus extension in D24 Rev on its biological functions.

### Longer V1/V2 and/or additional N-linked glycans probably govern expanded coreceptor use of D17 and D24

The two subtype C molecular clones reported here, D17 and D24, are unique in that they are capable of using a wide range of coreceptors including CCR5 and CXCR4 (Figure 6A). Phylogenetic characterization and Simplot analyses of the viral sequences (Figure 3D) ruled out the possibility of inter-subtype recombination in any of the viral gene segments. To examine if the expanded coreceptor use observed in this single donor can be largely attributed to HIV-associated dementia in the subtype C infection, we isolated biological viruses from three additional donors diagnosed with HIV-1 associated dementia in the absence of detectable opportunistic infections of the brain. All the three viruses used only CCR5 and were incapable of using any other coreceptor (see Additional file 2A). However, the assay used to determine coreceptor use in HOS derivatives may not be sensitive enough to detect minority viruses with different coreceptor requirement. Additionally, the possibility of X4 viruses emerging at a later stage of the infection cannot be ruled out. Development of dementia [67] and emergence of X4 viruses [12,13] are both manifested in advanced stages of the viral infection.

In subtype B infection, the molecular features of the Env that govern coreceptor specificity have been extensively analyzed. Although the V3 region of the env primarily determines coreceptor usage in subtype B [68], changes in V3 alone are not sufficient to alter the phenotype [44]. Furthermore, changes in other regions of the env, including the V1 and V2 [48-51], V4 [52] or C4 [49] have been shown to influence the coreceptor requirement in conjunction with V3. In subtype B infection, length polymorphism, especially insertion of additional amino acid residues in V1, V2 and V4 domains, has been associated with expanded coreceptor use [68]. In addition to length polymorphism, the degree of N-linked glycosylation in V3 and V1, V2 and the charge of the V3 loop appear to significantly influence coreceptor requirement of the viral strains [69,70]. The V3 loop of a vast majority of HIV-1 subtypes contains a single glycan, removal of which is strongly associated with the loss of CCR5 but gain of CXCR4 use [69]. Additionally, in the natural infection of subtypes A, B, D and A/E, a positive correlation was identified between increasing positive charge of the V3 loop and loss of the V3 glycosylation site both of which are expected to promote CXCR4 usage [25,69]. Such a correlation was not only absent in subtype C infection but also the total positive charges of V3 typically did not exceed +5 [47,57] suggesting that subtype C viruses should restrict coreceptor specificity preferentially to CCR5. It is noteworthy that a genetic analysis of env sequences of a few sero-positive infants failed to identify a correlation between the potential N-linked glycosylation sites and disease progression [26].

Given that both the clones D17 and D24 can make use of coreceptors other than CXCR4 and CCR5, it is interesting to examine if this phenomenon could have a pathological significance in vivo. Can viral clones of subtype C exhibiting expanded coreceptor use make use of other coreceptors on primary cells for infection in the absence of CCR5 and/or CXCR4? Long term virus culture in the presence or absence of inhibitor molecules or antibodies could possibly throw some light on this question. Such experiments in subtype B, however, demonstrated that virus strains which can use a broader range of coreceptors, failed to make use of coreceptors other than CCR5 and CXCR4 in primary cells [68].

With a total of 31 potential N-linked glycosylation sites, gp120 of D24 contains a potential for heavy glycosylation as compared to the three reference subtype C molecular clones (Figure 6C) or the three biological viruses isolated from subjects with dementia (see Additional file 2B). Many subtype C viruses contained as many as 27 glycans in gp120 while the MJ4 strain as few as 23 sites. The additional glycosylation sites in D24 were distributed among V1, V2 and V4 domains. Experimental evidence suggests a positive correlation between the magnitude of glycosylation and the capability of SF162, an R5 subtype B molecular clone, to use CD4 and CCR5 on the target cell [71]. N-linked glycosylation at certain residues is believed to enhance affinity of the env for the cellular receptors for HIV-1 [53,72] and HIV-2 [73]. Most, if not all, potential glycosylation sites in gp120 are utilized for carbohydrate attachment [74]. However, the relative contribution of each of these glycans for the viral infection and cell tropism is controversial [75]. Although the exact number of the potential glycosylation sites actually modified in vivo needs to be determined in D17 and D24, the heavy glycosylation potential of these clones probably enables the virus to efficiently infect the target cells especially in the brain where expression of CD4 and coreceptors is expected to be limiting [76]. Presence of env variants with higher affinities for CD4 and CCR5 was demonstrated in the brain compartment [77]. Although the molecular clones D17 and D24 were isolated from the peripheral blood, not from the brain, a dynamic exchange of viruses between these two compartments cannot be ruled out [78]. Heavy glycosylation is, thus, not only helpful in evading the immune surveillance [79] but also in leading to efficient viral infection [71]. The relative contribution of additional glycosylation sites identified in D24 env and the amino acid insertions in V1, V2 and V4 contributing to expanded coreceptor use of this viral isolate needs further investigation. Presently, we are generating chimeric D24 variant viruses by transferring individual V1/V2, V4 and C4 domains from the CCR5-using Indie-C1 molecular clone to corresponding regions of D24 env. Chimeric D24 viruses with single or combinations of Env variations will be examined for the coreceptor preference to gain more insights into the mechanisms of coreceptor-requirement in subtype C infection.

### Coreceptor switch: a universal phenomenon regardless of the subtype nature?

The association between coreceptor use and disease progression of HIV-1 is not completely understood. Viral isolates capable of using CXCR4 emerge in nearly half the infections of subtype B towards the advanced stages of the disease [12,13]. Why only R5 viruses can initiate a fresh infection and why in a significant number of subtype B infections, the viruses switch coreceptor use from CCR5 to CXCR4 in the advanced stages of the disease remains enigmatic [80]. In contrast to this situation of subtype B infection, a near absence or low incidence of coreceptor switch in the subtype C infection, even in the advanced stages of the disease, adds an additional layer of complication [9,19,20,22,24,25]. If the emergence of the X4 virus is essential for disease progression, as is seen in subtype B infection and in the case of other viral subtypes, the absence of coreceptor switch in the subtype C infection could be suggestive of the relatively less pathogenic nature of subtype C. In contrast to this assumption, subtype C isolates are responsible for the largest number of infections in the world [2]. Furthermore, studies on the natural history of the subtype C infection, especially from the African subcontinent [25,81,82] and India [83,84], fail to demonstrate significant difference in disease progression between subtype C and other subtypes although the data are incomplete or controversial [85], suggesting that subtype C isolates are probably as virulent as other viral subtypes. Thus, although a school of thought proposes that the less virulent and highly infectious nature of subtype C underlies its rapid global expansion [7], the experimental evidence in support of this hypothesis is scanty and even contradictory. Additionally, the viral load in subtype C infection does not appear to be different as compared to that of other subtypes [86,87]. Nevertheless, the preference for CCR5 by subtype C strains cannot be contested. A small number of studies, however, reported detection of dual-tropic and X4 viruses in a small but considerable number of subtype C infections by focusing on the advanced stages of the disease [8,21-26,54,88]. Biological viruses isolated from such subjects were capable of utilizing CCR5 and/or CXCR4. It is tempting to speculate that emergence of X4 viruses is a general phenomenon in a majority of the viral infections regardless of the subtype nature, however, the relative proportion between R5 and X4 viruses could be variable and unique for a given viral subtype. Detection of the presence of X4 viruses in a sample is probably limited by the sensitivity of the detection technique employed, especially when the X4 viruses are in a minority for detection, but not for disease progression. Most of the reporter cell lines routinely used for coreceptor phenotyping of the viral isolates are of non-T cell lineage engineered to express CD4 and one of the several coreceptor molecules. Cells of non-T cell lineage probably lack a potential to adequately support the growth of the minority viral variants present in the biological sample with different coreceptor requirement.

## Conclusion

In summary, we have constructed two infectious molecular subtype C clones, D17 and D24, from one Indian subject with HAD. The cloning strategy described here could have a broader application to other HIV-1 subtypes including N and O groups, HIV-2 and SIV. Both the clones possess several unique molecular properties including NF-κB site variation in the LTR and non-truncated Rev. We also detected the coexpression of two length isoforms of Rev in the donor proviral compartment and plasma viral pool. The viruses generated by the molecular clones D17 and D24 demonstrated expanded coreceptor use that is possibly correlated to sequence insertions in V1/V2/V4 and generation of additional N-linked glycosylation sites. The present study documented that molecular features of env that underlie expanded coreceptor use in subtype C infection are identical to what has been reported with the subtype B infection. Most studies of HIV-1 coreceptor requirement have relied on subtype B. The molecular clones reported here could be important tools in elucidating the genetic determinants that underlie HIV-1 subtype C neuropathogenesis, as well as unraveling the mechanisms of coreceptor preference in subtype C infection.

## Methods

### Clinical samples and virus cultures

Peripheral blood samples were collected from HIV-1 seropositive donors after obtaining informed consent and with the approval of the Institutional Bioethics Committees of Jawaharlal Nehru Centre for Advanced Scientific Research (JNCASR) and the National Institute of Mental Health and Neurosciences (NIMHANS). The donor (BL94/03), from whom the molecular clones were isolated, was a 39 year old male admitted in 2003 to the NIMHANS, Bangalore, India with clinical symptoms of potential HIV infection. The donor was a chronic alcoholic with a history of pulmonary tuberculosis. At the time of blood collection in 2003, he was drug-naïve for anti-retroviral therapy. The magnetic resonance imaging of the brain identified only frontal atrophy and no evidence of basal exudate or cortical enhancement thus excluding the possibility of meningeal inflammatory pathology. The serum and CSF samples were confirmed negative for mycobacteria (antibody, antigen and immune complexes), cerebral toxoplasmosis, cryptococcosis, cysticercosis and several other endemic bacterial and viral infections. Both the serum and CSF samples, however, were seropositive for HIV-1. Using subtype-specific PCR [63], the virus was determined to be a subtype C.

Mononuclear cells (PBMC) were isolated from blood by density gradient centrifugation using Ficol-Hypaque (1.077 g/ml, H8889, Sigma, St. Louis, Missouri, USA). Cells were mixed with equal number of PBMC isolated in a similar fashion from seronegative healthy donors 3 days earlier, CD8 cell depleted and mitogen-activated (5 μg of phytohemaglutinin/ml). Cell pool (2 × 10^6^/ml) was propagated in RPMI 1640 medium (Sigma) supplemented with 10% heat-inactivated fetal bovine serum (FBS, Biological Industries ltd. Kibbutz beit haemek, Israel), interleukin-2 (IL-2, 10 U/ml), penicillin (100 U/ml), streptomycin (100 mg/ml), and L-glutamine (2 mM). Fresh mitogen-activated donor PBMC were added to the cell pool when necessary. Production of HIV p24 was monitored from the culture supernatant twice a week using a commercial kit (Perkin Elmer Life Sciences, Boston, MA, USA).

### Amplification and cloning of the viral molecular clones

Genomic DNA was extracted from cells using the GenElute™ Blood Genomic DNA Kit (Sigma). Using two different sets of primer pairs (Table 2) and the nested-PCR approach, the provirus was amplified in two unequal fragments, a 3.0 kb fragment extending from the 5' LTR through the RT region and a 6.5 kb fragment spanning the rest of the provirus until the end of the 3'-LTR. Primers without nucleotide overhangs were used in the first-round and those with restriction sites in the second-round of amplification (Figure 1). The following amplification conditions were applied for both the fragments for both rounds of amplification: 94°C for 1 min, 65°C for 1 min, 68°C for 4 min for the first two of the total 30 cycles of the amplification. For the subsequent cycles, only two amplification steps were used 94°C for 30 sec for melting and 68°C for annealing. Starting from the third cycle an increment of 30 seconds was added to the extension step after every 5 cycles. The final 13 cycles were extended for 6 min each. We used Expand Long template polymerase mix with a proof-reading activity for the amplification (Roche, Alameda, CA). The amplification reaction of 50 μl contained 1 μg of genomic DNA, 250 μM of each of the four dNTPs, 30 pM of each of the primers, 2.75 mM of Mg^2+ ^and 1.25 U of enzyme mix. From the first-round amplification, 5 μl of the reaction mixture were transferred to the second-round amplification to serve as template. The 3.0 and 6.5 kb fragments were column-purified and digested with NotI plus MluI and MluI plus SbfI, respectively. We engineered NotI and MluI restriction sites into the multi-cloning site of a commercial vector pNEB193 (New England Biolabs, Beverly, MA) upstream of the existing SbfI site. The 3.0 and 6.5 kb fragments digested with appropriate restriction enzymes were incubated with the vector restriction digested at NotI and SbfI sites, in a single ligation reaction, followed by transformation using Stbl2 high-efficiency chemically competent *E. coli *cells (Invitrogen, Carlsbad, CA).

**Table 2:** Oligo-nucleotide primers for the viral amplification

**Amplification**	**Primers**	**HXB2 Coordinate**	**Sequence (5'-3')**
**N-term 3.0 kb fragment**			
First round	N558F	0001	TGGAAGGGTTAATTTACTCTAAGGAAAGGCAAGAGATCCTTG
	N453R	4820	CTATTATGTCTACTATTCTTTCCCCTGCAC
Second round	N533F	0001	GGGGGGCAGTGCGGCCGCTGGAAGGGTTAATTTACTCYMAGAAAAGRCAAG
	N502R	2878	TGAAAAATACGCGTCMCCCACATCCAGTACTGT
**C-term 6.5 kb fragment**			
First round	N501F	1851	CCAYAAAGCACGCGTKTTGGCTGARGCAATGAG
	N476R	9719	TGCTAGAGATTTTCCACACTGACTAAAAGGGTCTGAGGTCTCTAGTT
Second round	N503F	2841	ACTGGATGTGGGKGACGCGTATTTTTCAGTTCC
	N600R	9719	GCTTCGCATCCTGCAGGTGCTAGAGATTTTCCACACTGACTAAAAGGGTC

Sequences of the reverse oligo-nucleotides have been presented as inverse complement. Restriction enzyme sites have been underlined.

### Cells and cell lines

Human epithelial kidney 293T (HEK293T) cells were propagated in Dulbecco's modified eagle medium (DMEM) supplemented with 10% FBS, 2 mM glutamine and antibiotics. HOSCD4 parental cells and derivative cells expressing a wide range of chemokine receptors including CCR1, CCR2B, CCR3, CXCR4 or CCR5, were obtained from NIH AIDS Research and Reference Reagent Program (Rockville, MD). HOSCD4 parental cells were maintained in DMEM supplemented with 10% FBS and the derivative cell lines in medium containing 1 μg/ml of puromycin. Expression of CD4 and different coreceptors was confirmed by flow cytometry. MAGI cell lines expressing CCR5 and CXCR4 (NIH AIDS Research and Reference Reagent Program), were cultured in a similar fashion. For viral growth kinetics, we used CN-2 cells, *Herpesvirus saimiri*-immortalized human primary T-cells, and cultured them as described [43]. An astrocytoma neuronal cell line, GO-G-CCM, was obtained from National Center for Cell Sciences (NCCS, Pune, India) and cultured in medium containing DMEM and Hams F10 at a ratio of 1:1 and supplemented with 10% FBS. PBMC and primary monocytes were from anonymous HIV-1 negative donors. Monocytes were isolated by two rounds of density gradient centrifugation essentially as described previously [89]. CD8^+ve ^cells were depleted from the PBMC using a commercial kit (Cat. No. 15663, Rosette Sep, Stemcell Technologies, USA) and the cells enriched for CD4+ve marker were cultured in RPMI 1640 supplemented with 10% FBS, 5 μg phytohemaglutinin/ml for 72 h prior to infection. Monocytes were maintained in the same medium with 10 U/ml of IL-2 for 48 h prior to infection. Both PBMC and monocytes were seeded at 2 × 10^6^/well in 24-well plates and were propagated in 1.0 ml of medium.

### Production of viral stocks from the molecular clones

HEK293T cells were transiently transfected with recombinant viral clones using standard calcium phosphate protocol [90]. Cells seeded at low confluency in 6-well plates were transfected with a total of 3.0 μg of plasmid DNA, consisting of 2.7 μg of the viral plasmid vector and 0.3 μg of a CMV-β-galactosidase expression vector. The latter was included in all the transfections to serve as an internal control for transfection efficiency. Culture supernatants were harvested at 72 h, passed through 0.22 μ filter and stored in multiple aliquots in a deep freezer. The p24 concentration in each of the viral stocks was evaluated using a commercial ELISA kit. The TCID_50 _titers of the viral stocks were determined by using two subsets of MAGI cell lines expressing CCR5 and CXCR4 and serial fold dilutions of the viral stocks essentially as described [91]. Briefly, MAGI indicator cells were seeded at 0.5 × 10^5 ^cells per well in 12-well plates in complete DMEM supplemented with appropriate selection drug (G418, hygromycin and/or puromycin at final concentrations of 0.2 mg/ml, 0.1 mg/ml and 1 μg/ml, respectively). After 24 h, the cells were infected with the virus by replacing the culture medium from each well with 300 μl of appropriately diluted viral stocks (a serial 4-fold dilution) in complete DMEM supplemented with 10 μg per ml of polybrene but lacking selection drugs. Following 2 h of incubation of the cells at 37°C, 1.5 ml of complete DMEM were added to each well, and the cells were incubated for 2 days at 37°C in the presence of 5% CO_2_. To examine β-galactosidase expression, on day 3, the culture medium in each well was replaced with 1.0 ml of the fixing solution (1.0% formaldehyde and 0.2% glutaraldehyde in PBS) and the plates were incubated for 5 min at room temperature. The cells were washed two times with PBS, 500 μl of freshly prepared β-galactosidase staining solution (4 mM potassium ferrocyanide, 4 mM M potassium ferricyanide, 2 mM MgCl_2_, and 1 mM X-gal in PBS) was added to each well and the plates were incubated for 2–3 h at 37°C. Plates were washed twice in PBS and blue-stained cells were counted manually under low-resolution microscope. TCID_50 _value of each viral stock was calculated by multiplying the cell-count with the dilution factor.

### Reporter gene analysis and viral infection assay

In experiments to determine expression of Tat from the molecular clones, we cotransfected HEK293 cells with 9 μg of the viral plasmid and 1 μg of a bicistronic reporter vector using the calcium phosphate transfection protocol [90]. In the reporter vector, two different genes, secreted alkaline phosphatase (SEAP) and enhanced green fluorescent protein (GFP) were placed under the control of a subtype C LTR [64]. Expression of the reporter genes was monitored at 24, 48 and 72 h post-transfection. A reference subtype C molecular clone Indie-C1 served as the positive control and the vector backbone as the negative control. Cell-free culture supernatant was harvested 72 h post-transfection and monitored for viral p24 production. SEAP assay was performed essentially as described previously [89]. GFPexpression was visualized 24–72 h after transfection under UV and images were captured at 20× magnification using a digital camera (DFC320, Leica, Heerbrugg, Germany) and processed with Image Manager software (IM50, Leica).

### Western-blot analysis of the viral antigens

Virus particles were pelleted from 10 ml of fresh or frozen culture supernatants by high-speed centrifugation at 70,450 g for 2.5 h at 4°C. The viral pellet was resuspended in lysis buffer (0.15 M NaCl; 0.05 M Tris-HCl, pH 7.2; 1% Triton X-100; 1% sodium deoxycholate and 0.1% sodium dodecyl sulfate). The total amount of protein obtained was quantified at 280 nm using the NanoDrop spectrophotometer (ND-1000, Wilmington, DE, USA). Ten μg of total protein for each sample was mixed with reducing buffer (0.08 M Tris-HCl, pH 6.8; 0.1 M dithiothreitol; 2% sodium dodecyl sulfate; 10% glycerol and 0.2% bromophenol blue), boiled for 3 min and resolved on an SDS-polyacrylamide gel electrophoresis (SDS-PAGE) gels with a linear gradient of 6 to 15% polyacrylamide. Resolved proteins were transferred onto PVDF transfer membrane (Hybond-P, Amersham Biosciences, UK), using semidry transfer apparatus (Hoefer TE77, Amersham Biosciences), at 50 V for 1 h. Viral proteins were visualized by immunoblotting with pooled sera from HIV-1-seropositive individuals infected with subtype C from Southern India.

### Determination of coreceptor usage

HOS cell lines with or without the CD4 receptor and a broad range of chemokine receptors including CCR1, CCR2B, CCR3, CXCR4 and CCR5 were used. Viral stocks, 500 TCID_50 _units each, were added to cells in 24-well plates in DMEM medium supplemented with 10% FBS. After 16 h of incubation, residual virus was washed off and the cells were incubated in complete medium. Culture supernatants were sampled periodically up to day 22 and viral infection of the target cells was monitored using a p24 capture ELISA. The cells were split once a week. To determine the specificity of coreceptor usage, we incubated the target cells with specific inhibitors, TAK-779 [92] and bicyclam JM-2987 [93] (The NIH AIDS Research and Reference Reagent Program) for CCR5 and CXCR4, respectively. The cells, 1 h prior to the addition of the virus, were treated with respective inhibitor at different concentrations, TAK-779 at 0.1 through 100 μM and JM-2987 at 6 through 90 nM. The inhibitors were present in the culture medium at the indicated concentration through the infection process. In a similar fashion, recombinant soluble CD4 consisting of the D1 and D2 domains, at a final concentration of 1 μg/ml, to confirm CD4 use by the viruses. Additionally, we also used two different anti-human CD4 monoclonal antibodies, F2 and F4 (generated in our laboratory), at a final concentration of 0.5 μg/ml to block CD4-mediated infection of the viruses.

### Sequencing and sequence analyses

We designed more than 40 individual sequence primers spanning the entire length of the viral genome on both the strands. The primers target sequences highly conserved among diverse viral subtypes and placed approximately 500 nucleotides apart (see Additional file 3). Overlapping contiguous sequences, for both D17 and D24, were obtained throughout the genome using the dye-terminator chemistry. Sequencing reactions were performed on the plasmid DNA. Chromatograms from ABI377 and Beckman CEQ8000 sequencers were edited using Sequencher (GeneCodes, Ann Arbor, Mich.). Sequence contigs were assembled using Lasergene software (DNASTAR, Inc., Madison, WI, USA) to generate complete nucleotide sequences of the molecular clones. Partial DNA sequences of various viral gene segments of the other replication-defective molecular clones were obtained in a similar fashion.

The complete nucleotide sequence of the molecular clone D24 is available from GenBank under the accession number EF469243. The gp120 sequences of the four primary viral isolates used in coreceptor evaluation studies (Additional file 2B) are available from GenBank under the accession numbers EU492868–EU492871.

Sequences of the newly derived viral clones were aligned with full-length reference sequences of several Group M viruses obtained from the Los Alamos sequence database. Nucleotide sequences were gap-stripped and aligned using CLUSTAL X [94] and Neighbor-joining trees were generated with the Kimura 2 parameter substitution model by using MEGA3.2 software [95]. Pairwise evolutionary distances were estimated by using DNADIST from the PHYLIP 3.6 package [96], and the reliability of the topologies was evaluated by bootstrap analysis with 100 replicates by using SEQBOOT, DNADIST, NEIGHBOR and CONSENSE. To determine the recombinant nature of the isolated molecular clones, we performed bootscanning using the algorithm of maximum parsimony with a sliding window of 400 nucleotides overlapping by 20 nucleotides. SimPlot was used to plot similarity versus position. The following reference sequences of diverse viral subtypes were used in the analysis. Subtype A (A1.KE.94.Q23_17/AF004885, A1.SE.94.SE7253/AF069670, A1.UG.85.U455/M62320, A1.UG.92.92UG037/U51190, A1.UG.x.UG029/AB098332, A1.UG.x.UG029/AB098333, A1.UG.x.UG031/AB098330, A1.UG.x.UG031/AB098331, A2.CD.97.97CDKTB48/AF286238, A2.CY.94.94CY017_41/AF286237), subtype B (B.FR.83.HXB2-LAI-IIIB-BRU/K03455, B.FR.83.REHTLV3/X01762, B.FR.x.F12CG/Z11530, B.FR.x.NL43/M19921, B.US.83.RF/M17451, B.US.86.JRFL/U63632, B.US.86.YU-2/M93258, B.US.90.WEAU160/U21135, B.US.x.NL43xWC001/AF003887), subtype C (C.BR.92.BR025-d/U52953, C.BW.00.00BW147127/AF443091, C.BW.96.96BW0502/AF110967, C.ET.86.ETH2220/U46016, C.IN.93.93IN101/AB023804, C.IN.95.95IN21068/AF067155, C.ZM.x.HIV1084i/AY805330), subtype D (D.CD.83.ELI/K03454, D.CD.83.NDK/M27323, D.CD.84.84ZR085/U88822, D.UG.92.92UG001/AJ320484, D.UG.94.94UG114/U88824), subtype F (F1.BE.93.VI850/AF077336, F1.BR.89.BZ126/AY173957, F1.BR.93.93BR020_1/AF005494, F1.FI.93.FIN9363/AF075703, F1.FR.96.MP411/AJ249238, F2.CM.95.MP255/AJ249236, F2.CM.95.MP257/AJ249237, F2.CM.97.CM53657/AF377956), subtype G (G.BE.96.DRCBL/AF084936, G.FI.93.HH8793_12_1/AF061641, G.FI.93.HH8793_1_1/AF061640, G.NG.92.92NG083/U88826, G.SE.93.SE6165/AF061642), subtype H (H.BE.93.VI991/AF190127, H.BE.93.VI997/AF190128, H.CF.90.056/AF005496), subtype J (J.SE.93.SE7887/AF082394, J.SE.94.SE7022/AF082395) and subtype K (K.CD.97.EQTB11C/AJ249235, K.CM.96.MP535/AJ249239).

### Statistical methods

All statistical analyses were performed with the SPSS package. Experiments were performed three or more times and values obtained from three replicate samples were averaged in each experiment. Data are presented as mean value with the standard deviation (± 1 S. D.). Statistical significance was tested using Student's paired *t*-test. Differences were considered significant at *p *< 0.05.

## Competing interests

The author(s) declare that they have no competing interests.

## Authors' contributions

PKD, NBS, MV AM and KKA performed the experimental work. AVM and PR did the sequence analysis. AM, PS, SKS counseled the donors and collected the clinical history of the volunteers. UR and VRP conceived of the study, participated in its design and coordination and wrote the manuscript. All authors read and approved the final manuscript.

## Supplementary Material

Additional file 1 Replication kinetics and Coreceptor usage of BL94 virus. **(A) **The ability of BL94 virus to propagate on PBMC. PBMC of donor BL94 were cocultured with CD8-depleted and mitogen-activated PBMC from a healthy donor. The production of p24 in the spent medium was monitored using a commercial antigen-capture ELISA kit. **(B) **Coreceptor phenotyping of the BL94 biological virus in HOS cell lines. HOS cell lines expressing CD4 and one of the several coreceptors including CCR1, CCR2B, CCR3, CXCR4 and CCR5 or CD4 alone or CCR5 alone were tested for viral infectivity. These cell lines were infected overnight with BL94 virus, residual virus was washed off and the cells were incubated up to 14 days. Secretion of p24 into the medium was monitored every third day using a commercial kit and data for day-7 are presented here. Data presented was representative of three independent experiments performed in triplicates. Click here for file

Additional file 2 Coreceptor phenotyping of subtype C biological viruses. **(A) **Profile of coreceptor use in HOS cell lines. Coreceptor usage of D24 virus was compared with that of biological viruses isolated from three demented and two non-demented subjects. The assay details are essentially as described in Figure 6A. **(B) **Amino acid sequence comparison of the gp120 extra-cellular variable loops and the C4 domain. The amino acid sequence of D24 env loops is aligned with those of two biological viruses isolated each from demented (A43 and BL95) and non-demented (S53 and BL43) seropositive subjects. Note that the env sequence of the third demented donor (BL96) was ommitted from the analysis as this virus was possibly an A/C recombinant in env. Dots indicate sequence homology and dashes gaps. Putative N-linked glycosylation sites have been highlighted by shaded boxes. Figures in the parentheses indicate the number of potential glycans in respective loops. Click here for file
