# Editorial Expression of Concern: Exceptional molecular and coreceptor-requirement properties of molecular clones isolated from an human immunodeficiency virus Type-1 subtype C infection

**DOI:** 10.1186/s12977-024-00654-x

**Published:** 2024-11-07

**Authors:** Prasanta K. Dash, Nagadenahalli B. Siddappa, Asokan Mangaiarkarasi, Aruna V. Mahendarkar, Padmanabhan Roshan, Krishnamurthy Kumar Anand, Anita Mahadevan, Parthasarathy Satishchandra, Susarla K. Shankar, Vinayaka R. Prasad, Udaykumar Ranga

**Affiliations:** 1 Molecular Virology Laboratory, Molecular Biology and Genetics Unit, Jawaharlal Nehru Centre for Advanced Scientific Research, Bangalore, India; 2 Department of Neurology, National Institute of Mental Health and Neurosciences, Bangalore, India; 3 Department of Microbiology and Immunology, Albert Einstein College of Medicine, Bronx, NY USA; 4 Dana-Farber Cancer Institute, Harvard Medical School, 44 Binney Street, JFB-809, Boston, MA 02115-6084 USA

The Editors-in-Chief are issuing this Editorial Expression of Concern to alert readers that a concern has been raised about some of the data reported in this article. Specifically, in Fig. 2a the image panels for D7 and D21 appear to overlap. Due to the age of this paper, the authors were not able to provide raw data for this figure upon request by the Publisher. Readers are therefore advised to interpret the data presented in this article with caution.

Udaykumar Ranga has stated on behalf of all authors that they agree with this Editorial Expression of Concern.

The original article can be found online at 10.1186/1742-4690-5-25.

