# Supplementary figures and images for: Exceptional molecular and coreceptor-requirement properties of molecular clones isolated from an Human Immunodeficiency Virus Type-1 subtype C infection
Source: Retrovirology. 2008 Mar 7;5:25. doi: 10.1186/1742-4690-5-25 (PMC2292743; doi:10.1186/1742-4690-5-25)

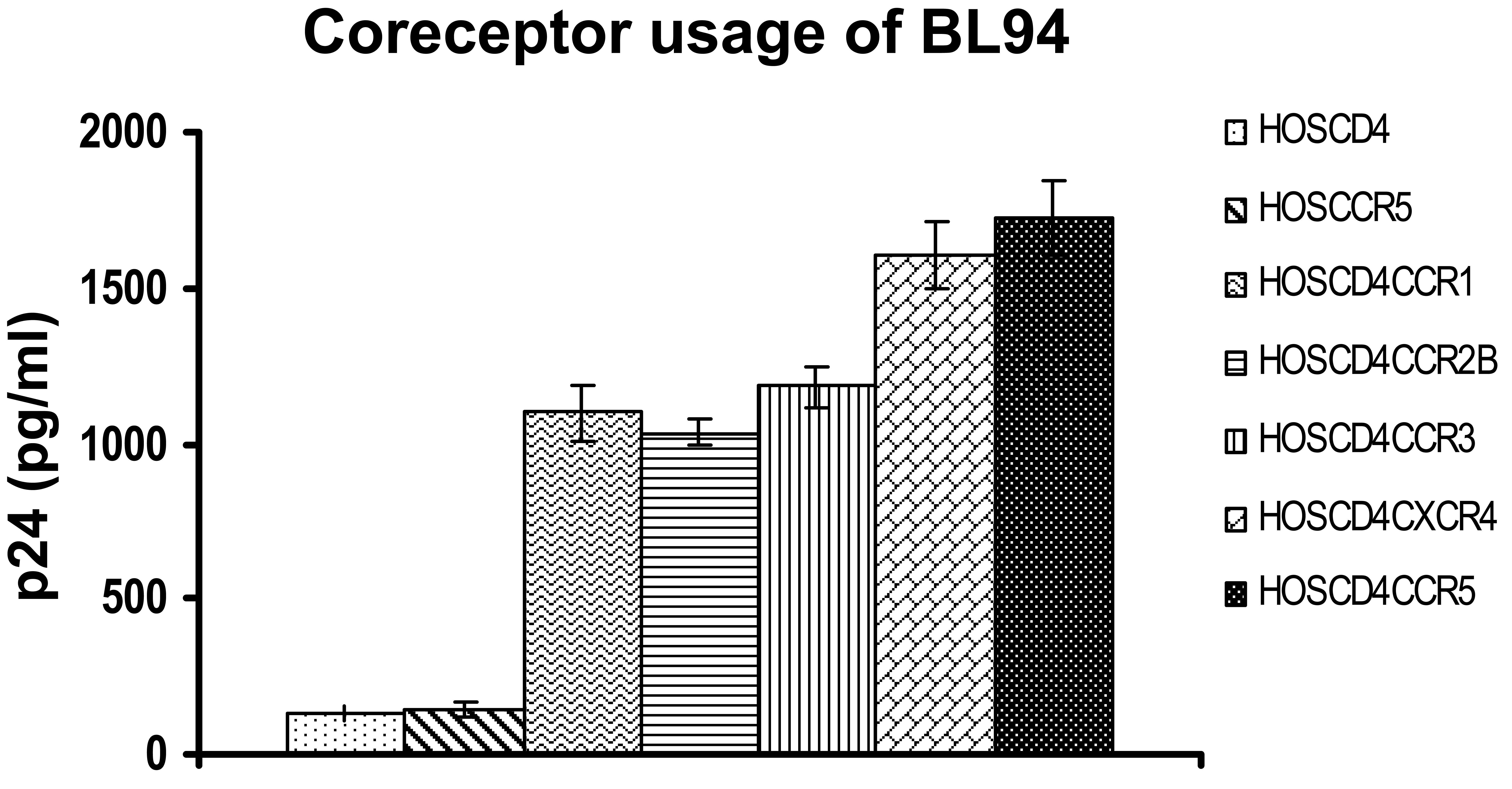

Supplement: Additional file 1 — Replication kinetics and Coreceptor usage of BL94 virus. (A) The ability of BL94 virus to propagate on PBMC. PBMC of donor BL94 were cocultured with CD8-depleted and mitogen-activated PBMC from a healthy donor. The production of p24 in the spent medium was monitored using a commercial antigen-capture ELISA kit. (B) Coreceptor phenotyping of the BL94 biological virus in HOS cell lines. HOS cell lines expressing CD4 and one of the several coreceptors including CCR1, CCR2B, CCR3, CXCR4 and CCR5 or CD4 alone or CCR5 alone were tested for viral infectivity. These cell lines were infected overnight with BL94 virus, residual virus was washed off and the cells were incubated up to 14 days. Secretion of p24 into the medium was monitored every third day using a commercial kit and data for day-7 are presented here. Data presented was representative of three independent experiments performed in triplicates. [file 1742-4690-5-25-S1.jpeg]

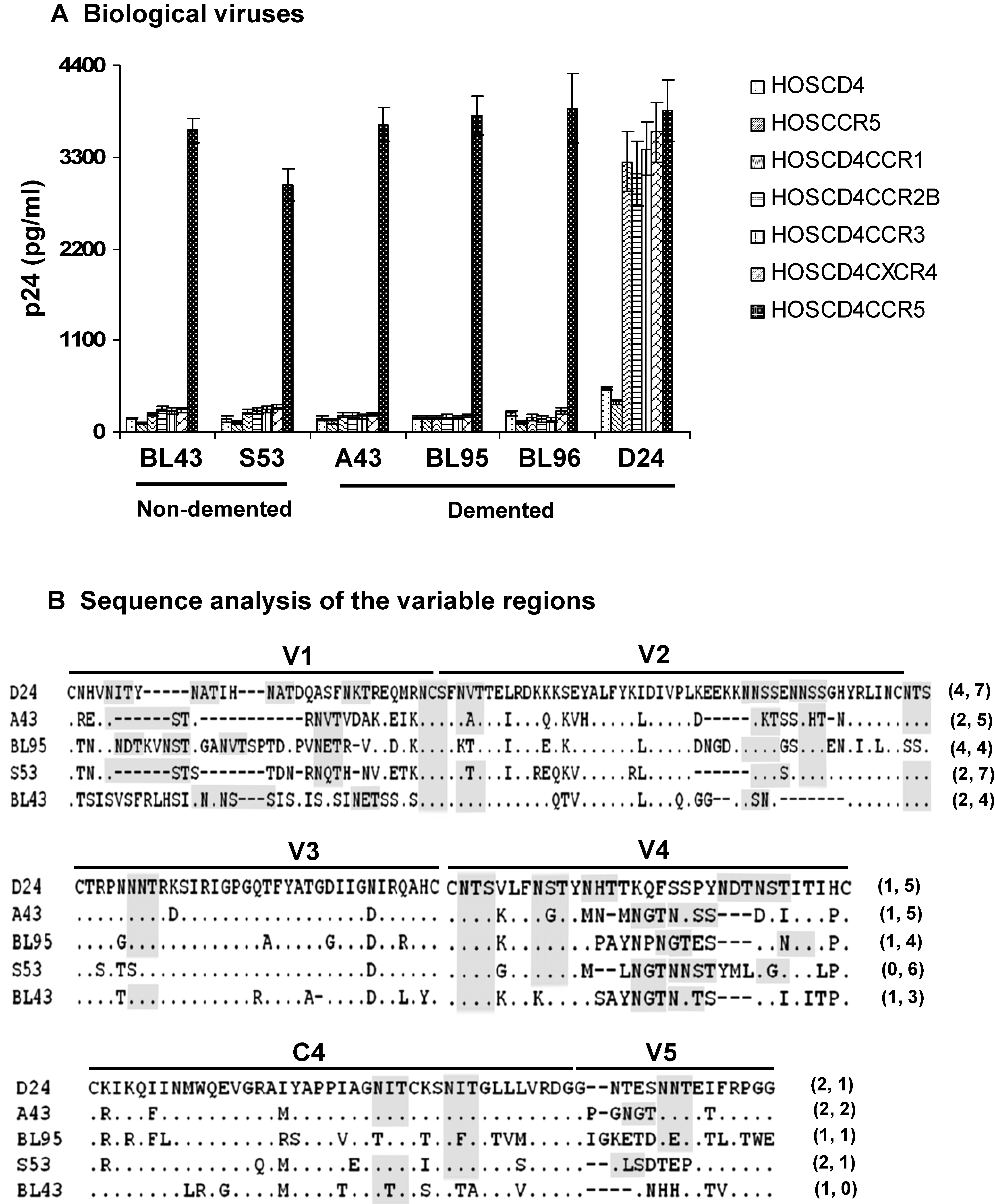

Supplement: Additional file 2 — Coreceptor phenotyping of subtype C biological viruses. (A) Profile of coreceptor use in HOS cell lines. Coreceptor usage of D24 virus was compared with that of biological viruses isolated from three demented and two non-demented subjects. The assay details are essentially as described in Figure 6A. (B) Amino acid sequence comparison of the gp120 extra-cellular variable loops and the C4 domain. The amino acid sequence of D24 env loops is aligned with those of two biological viruses isolated each from demented (A43 and BL95) and non-demented (S53 and BL43) seropositive subjects. Note that the env sequence of the third demented donor (BL96) was ommitted from the analysis as this virus was possibly an A/C recombinant in env. Dots indicate sequence homology and dashes gaps. Putative N-linked glycosylation sites have been highlighted by shaded boxes. Figures in the parentheses indicate the number of potential glycans in respective loops. [file 1742-4690-5-25-S2.jpeg]
